# Lessons learned from COVID-19 vaccine acceptance among pregnant and lactating women from two districts in Kenya to inform demand generation efforts for future maternal RSV vaccines

**DOI:** 10.1186/s12884-024-06425-y

**Published:** 2024-03-27

**Authors:** Rupali J. Limaye, Prachi Singh, Berhaun Fesshaye, Ruth A. Karron

**Affiliations:** 1 Department of International Health, Bloomberg School of Public Health, Johns Hopkins University, Baltimore, USA; 2 Department of Epidemiology, Bloomberg School of Public Health, Johns Hopkins University, Baltimore, USA; 3 Department of Health, Behavior & Society, Bloomberg School of Public Health, Johns Hopkins University, Baltimore, USA; 4 Bloomberg School of Public Health, International Vaccine Access Center, Johns Hopkins University, Baltimore, USA

**Keywords:** COVID-19 vaccine, Kenya, Maternal immunization, RSV vaccine

## Abstract

**Background:**

Respiratory syncytial virus (RSV) is a leading cause of acute lower respiratory infections globally, with most RSV-related deaths occurring in infants < 6 months of age. The highest burden of RSV is in low-and-middle income countries, and in sub-Saharan Africa, RSV may be responsible for almost half of all hospital admissions with severe or very severe pneumonia among infants under 1 year. There is a maternal RSV vaccine on the horizon. Our study objective was to better understand how lessons learned from the COVID-19 vaccine experience rollout among pregnant and lactating people in Kenya could inform future maternal RSV vaccine rollout.

**Methods:**

This qualitative study interviewed 16 healthcare providers including doctors, nurses, midwives, community health workers, and vaccinators. Participants were recruited from two counties in Kenya and included healthcare providers that served diverse communities. A grounded theory approach was used to analyze the data.

**Results:**

As healthcare providers interviewed were instrumental in COVID-19 vaccine rollout among pregnant women in Kenya, they provided lessons learned from the COVID-19 vaccine experience to inform future maternal RSV vaccine rollout. Community sensitization emerged as the most critical lesson learned, including communication, mobilization, and education. Using communication to ensure community awareness of RSV, community awareness of RSV harms and benefits of RSV maternal vaccines, and providing up-to-date, clear information about maternal RSV vaccines emerged as lessons. Related to mobilization, participants identified the need for healthcare providers and community leaders to gain the trust of communities, and the importance of routinizing the vaccine. Finally, for education, participants outlined critical questions patients would have about a maternal RSV vaccine, including those related to vaccine safety concerns, duration of protection, and vaccine dosing.

**Conclusions:**

This is one of the first studies that has examined how lessons learned from the COVID-19 vaccine rollout for pregnant and lactating women can inform the rollout of future maternal vaccines, including an RSV maternal vaccine. As healthcare providers are directly involved in vaccine rollout, their perspectives are crucial for successful vaccine acceptance.

## Background

Vaccination during pregnancy, or maternal immunization, is a key strategy to protect against maternal, neonatal, and infant disease [1, 2]. Maternal antibodies are passed through the placenta to the fetus, providing passive immunity for the first few months of the infant’s life, when they are the most susceptible to disease [3]. Several maternal vaccines already in use have been shown to effectively protect neonates and infants against tetanus, pertussis, and influenza, and there are new maternal vaccines for additional diseases currently under development [4, 5].

Respiratory syncytial virus (RSV) is a leading cause of acute lower respiratory infections (ALRI) globally, especially in children under five, with most RSV-related deaths occurring in infants less than 6 months of age in low- and middle-income countries [6]. The maternal RSVpreF vaccine has been shown to safely prevent severe RSV lower respiratory tract illness, lower the rates of RSV-associated hospitalization, and provide protection across the spectrum of RSV illness severity in infants up to 6 months of age [7]. This vaccine has now been licensed and approved for administration during pregnancy by several regulatory authorities, including the Food and Drug Administration (FDA) and the European Medicines Authority (EMA) [7].

While COVID-19 vaccines were the most recent recommendation for pregnant persons, they are not the first. Globally, tetanus toxoid-containing vaccines are the most widely vaccine recommended during pregnancy, with the majority of countries having a policy in place for maternal tetanus immunization [8, 9]. Despite long-standing efforts to eliminate maternal and neonatal tetanus, 18 low- and middle-income countries have yet to achieve elimination, and several countries are still not meeting tetanus toxoid-containing vaccine coverage targets [9, 10]. Barriers to optimal maternal vaccine uptake include health system issues, such as staff shortages and inadequate reporting systems [10]. However, healthcare provider (HCP) recommendation, effective communication between HCPs and beneficiaries, and HCP awareness of vaccination policies have also been shown to be important barriers to uptake [10]. Proactively compiling experiences prior to introduction of RSV maternal vaccines in settings such as Kenya may help to increase maternal acceptance and uptake. While the COVID-19 vaccine rollout process existed during a global pandemic, safety concerns due to the novelty of the vaccine led to increased hesitancy [11–13]. Given RSV vaccines are also newly developed, similar concerns will likely emerge during rollout.

Given the key role that healthcare providers play in rollout and uptake of new vaccines, it is essential to understand and learn from their experiences. Adequate communication and timely information dissemination from healthcare providers have been shown as determinants of maternal vaccine acceptance; therefore, preparing providers with information prior to RSV vaccine rollout may be beneficial [14, 15]. In this study, we interviewed healthcare providers from two counties in Kenya about their experiences with COVID-19 vaccine rollout to pregnant and lactating women to inform rollout of a future RSV maternal vaccine.

## Methods

In-depth interviews were conducted with 16 healthcare providers that included doctors, nurses, midwives, community health workers, and vaccinators. Participants were recruited from two counties, with two communities in each county: Nakuru (rural), and Mombasa (urban). Ten sites in each county (18 public hospitals and 2 private hospitals) were used for recruitment and the sample was evenly split between urban and rural sites. As this was an exploratory study, we planned to interview healthcare providers until data saturation was reached.

Data collection occurred in August–September 2022, during which COVID-19 vaccine rollout among the general population and for pregnant and lactating people was ongoing. Data collectors participated in a three-day training and completed an online human ethics training. Data collectors consulted with charge nurses at health facilities to determine which healthcare providers on duty worked with pregnant and post-partum people. In smaller health facilities, every provider working with pregnant or post-partum people was approached for recruitment. In larger facilities, nth sampling was used, with n varying based on the size of the facility. Participants met inclusion criteria if they were at least 18 years of age and able to give informed consent. Oral informed consent was obtained. Participants were asked about their experiences related to maternal vaccination broadly, including vaccinating pregnant and lactating people against COVID-19, as well as influences related to maternal vaccination acceptance in the communities they served. Interviews were conducted in English, Swahili, or other local languages as necessary in a semi-private setting. All interviews were audio recorded, transcribed, and translated into English by members of the study team and external translators. All audio recordings were stored on encrypted servers, and only members of the study team had access to the data.

A team of 6 used a grounded theory approach and data were managed using Atlas.ti (version 23). We used purposive sampling to include a variety of healthcare providers. Data were collected in August–September 2022 and interviews were transcribed. The code list was then developed, refined, and finalized over three rounds of open coding. Following agreement of a code list, the team coded the transcripts, holding discussions on emerging themes after coding 50% of the transcripts. Two members of the team conducted inter-rater reliability with ~ 10% of the transcripts that neither of them had coded (3 transcripts). Reliability was 89%. The team then identified themes and sub-themes that emerged from the data to identify key lessons learned. This study received ethical approval from (*Kenya Medical Research Institute*) and (*Johns Hopkins Bloomberg School of Public Health Institutional Review Board*).

## Results

**Fig. 1:**
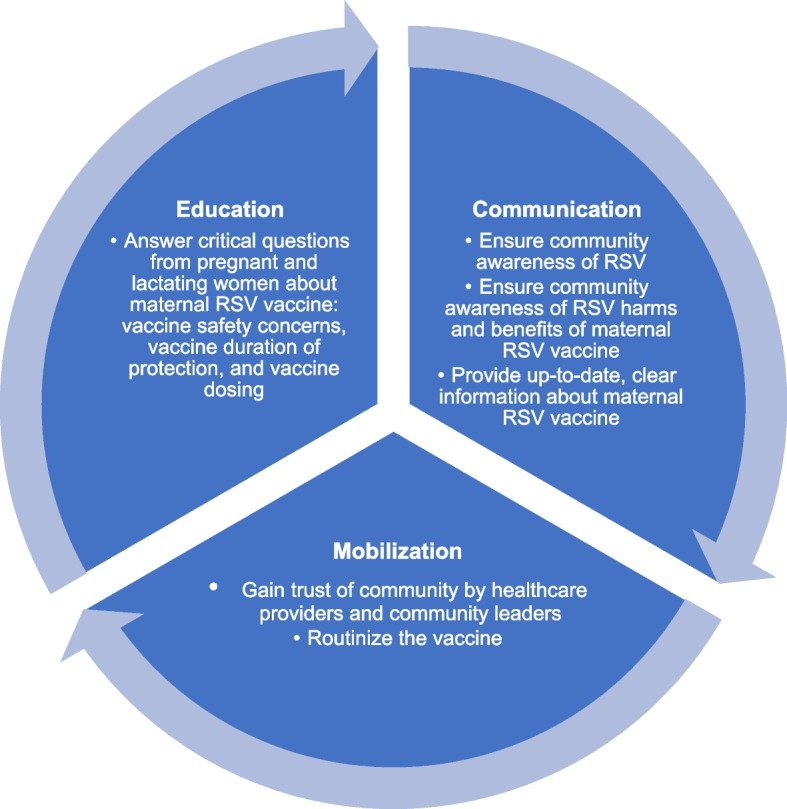
Community sensitization components for future maternal RSV vaccine acceptance among pregnant and lactating women

A total of 16 healthcare providers were interviewed. No minors or lower-literate individuals were involved in the study.Given that COVID-19 vaccine rollout was occurring during the time of data collection, including among pregnant and lactating persons in Kenya, healthcare providers provided lessons learned from the COVID-19 vaccine experience rollout to inform future maternal RSV vaccine rollout. The most prevalent lesson that emerged was related to community sensitization broadly, including communication, mobilization, and education specifically. While communication, mobilization, and education can overlap, we sought to articulate each domain based on the results (Fig. 1). For communication, participants identified using communication to ensure community awareness of RSV disease, of the potential harms and benefits of RSV maternal vaccines, and provide up-to-date, clear information about maternal RSV vaccines. Related to mobilization, participants identified the need for healthcare providers and community leaders to gain the trust of community members and making the vaccine a routine part of prenatal care. Finally, for education, participants outlined critical questions that should be answered which included vaccine safety concerns, duration of protection, and vaccine dosing.

### Community sensitization regarding future maternal RSV vaccines: communication and mobilization

Healthcare providers stressed the need for communities to know about RSV disease generally, as articulated by this healthcare provider from Nakuru: “*The communities need to understand first. What is this RSV? Just basic knowledge. Then you will need to explain to them that the cough you’ve been having, the chest pains, is this virus and we are intending to give you a vaccine to prevent all these problems. They need to understand why and what is this RSV first*.” (Kamara Dispensary, Nakuru). In addition to basic awareness about RSV disease, this healthcare provider from Mombasa believed that RSV vaccine acceptance would be easier if the community was able to see the harms of RSV as well as the benefits of a maternal RSV vaccine. This was because the community was able to see the negative effects of COVID-19 disease and the positive benefits of the vaccine: “*With the lessons learnt with COVID-19—and the fact that the RSV vaccine is coming post-COVID, I think it will be an easier time for community to embrace an RSV vaccine because we saw how COVID killed people and this is another disease affecting the respiratory trachea—this gives the seriousness of the vaccine to the people. The healthcare workers (will accept an RSV vaccine) because this is a disease that is affecting our breathing system and this is another disease like COVID which was very serious—as it was killing people in masses. I’m seeing that in the future the RSV vaccine being more acceptable than the other previous vaccines that were introduced, provided that people will know the benefits, what the disease is, and if taking the vaccine, will it completely give proper immunity from this disease*.” (Tudor Sub-County Hospital, Mombasa). Additionally, this point about whether a new vaccine would ‘give proper immunity’, i.e., prevent infection of the disease also points to the importance of communication and education about what the vaccine can do to manage expectations.

In addition to understanding the harms and vaccine benefits, clear information about the safety of the vaccine was crucial for COVID-19 vaccine uptake among pregnant and lactating people. As such, healthcare providers suggested ensuring that there was up to date and accurate information related to the safety of maternal RSV vaccines. Accurate information was of particular importance for successful uptake, as this healthcare provider relayed how safety of the COVID-19 vaccine changed over time, and how this affected attitudes and uptake among pregnant and lactating women: “*Now, one thing I remember when we started rolling out this COVID vaccination to everyone, the first… there was information that was available that said it is not safe to pregnant women. That information actually affected everything. So pregnant women seeking the vaccine were very low. Actually I remember teachers were prioritized for the vaccine, and pregnant teachers did not take the vaccine. Because the information was that it is not safe for them. I think the information that is available guides us and guides the pregnant women seeking that vaccine. So if this new RSV vaccine will come with information that it is safe in pregnancy, I think pregnant women will take it. There are still those pregnant women that are resistant*.” (Kiptangwanyi Health Centre, Nakuru). This healthcare provider from Mombasa still perceived resistance to COVID-19 vaccines among pregnant women in the community they served: *“The community has not embraced the COVID vaccine in pregnant mothers. The reason is that there are misconceptions which are around, and so the community has not embraced COVID vaccines. The community—they have not accepted that this vaccine is safe because they still believe the vaccine has not been tried well and so the pregnant mothers are hesitant even now.*” (Mlaleo Health Centre, Mombasa). This healthcare provider from Mombasa reflected upon the need for wide dissemination of information related to vaccine safety to communities given the COVID-19 experience: “*For now, I can say that there are fewer concerns about COVID vaccines compared to the first time that they introduced this vaccine. The first time—that’s when rumors were going that the vaccine should not be given to pregnant women. But now, people have enough knowledge, we have seen a lot of women here after you have finished with their appointment they ask for the covid vaccine, they ask where can I get my booster. And now because the first people were lacking that knowledge about the vaccine but now everyone is enlightened about it and they are ready to go and take the vaccine*.” (Naivasha Sub-County Hospital, Nakuru). This healthcare provider articulated how healthcare providers themselves first believed that the COVID-19 vaccine was unsafe, but this concern was allayed over time with up-to-date accurate information, and how community word-of-mouth was so powerful for acceptance: “*Initially there was that worry even among the healthcare workers that the COVID vaccine was unsafe, but when they provided information about its safety and people were educated about it, and were told that now it is safe, actually at the community level, I had people calling when they are pregnant, that they now want vaccination. One community member can spread word to so many*.” (Tudor Sub-County Hospital, Mombasa). Given the concerns among healthcare providers, this healthcare provider from Mombasa pointed to the importance of healthcare providers being sensitized first with the most complete information, so that they could subsequently recommend a new vaccine to their communities: “*If recommended by the MOH, that means that they have consulted WHO and it is safe for our mothers, so I think I would recommend that the community to be aware of it. But us healthcare workers should be first to be sensitized on the RSV vaccine, and know about it, meaning knowing the benefit, what it is, how is it given, what is the dose, how many doses, where it is given*.” (Tudor Sub-County Hospital, Mombasa).

This healthcare provider from Nakuru asserted the importance of patience and identified building trust as critical for COVID-19 vaccine acceptance: “*It was not easy to persuade pregnant women to get the COVID vaccine, it wasn’t easy. We had to do a lot of health education, inform them more on the risks, talk to them when they come for the first visit. Even if they first do not accept, just continue, have the patience, talk to them, talk and talk, it took so much, it was not easy. But finally, they accepted. But if one accepts, you have already won. They were really afraid that they could miscarry, or have babies without limbs and such things. But when we managed to vaccinate one, she became a champion. Slowly trust was built. So now at least they have the knowledge and ask for it.*” (Kamara Dispensary, Nakuru). Trust was also the reason why community members sometimes listened to community leaders over healthcare providers in the community this healthcare provider from Mombasa worked in: “*My role is very crucial for new vaccine uptake like RSV, especially to the community to accept and having had the experience of doing several campaigns on vaccinations of polio vaccine, tetanus vaccine, the community has its own perceptions especially when it comes to vaccines. We did a campaign on tetanus but the turnout was very low because the community listens to and trusts its leaders more than the healthcare workers*.” (Tudor Sub-County Hospital, Mombasa).

Finally, healthcare providers alluded to including any new vaccine in the routine vaccine schedule for successful acceptance, including future maternal RSV vaccines. This healthcare provider from Mombasa referenced the routinization of tetanus vaccination during pregnancy as an essential factor for maternal tetanus vaccine acceptance and as an important lesson for future maternal vaccine acceptance: “*Pregnant women were able to come for the normal tetanus vaccination. It was simple because it was a normal routine vaccine, and the pregnant women already were given information and the importance of it. That is why they were not reluctant about accepting it*.” (Port Reitz Sub-County Hospital, Mombasa). This healthcare provider from Nakuru also referenced routinization of vaccines, and how a vaccine for pregnant women specifically will be easier for pregnant women to accept, in comparison to COVID-19 vaccines: “*You know COVID was for everybody but if you introduce a vaccine for the… mothers only—that one is different. Because when pregnant women come to the facility we tell them there is a new vaccine for this specific disease, and we are giving it to you at this gestation period. They will have no option but to accept it. But COVID was different because it was for everybody*.” (Bahati Rural Health Centre, Nakuru).

### Community sensitization regarding future maternal RSV vaccines: education to answer questions about future maternal RSV vaccines

Healthcare providers identified vaccine safety as the primary concern pregnant and lactating women would have about a maternal RSV vaccine. These concerns included how the vaccine could potentially affect the unborn child, infant, and mother. Per this healthcare provider from Nakuru: “*The pregnant women will first be worried about the potential side effects from the vaccine, and the lactating mothers—almost all of them will ask if it will affect the child, and will it affect breastfeeding. Maybe some will say that when you take a certain vaccine that there is that reduction of breast milk. So I think they would ask those questions.”* (Naivasha Sub-County Hospital, Nakuru). This healthcare provider from Mombasa had similar feedback: “*Women will ask—does (the RSV vaccine) have teratogenic effects which could affect my child negatively? Those pregnant mothers will ask, is it going to make me sick? Because most of those immunizations give low grade fevers, the malaise and the chills. Will the RSV vaccine have those side effects? Or the breastfeeding mothers, they have those worries of leaking through the breast to the child, they will ask—is it going to affect my child negatively or positively? Those are the big worries.”* (Likoni Sub-County Hospital, Mombasa). Besides these concerns, healthcare providers also raised the potential effects of the vaccine on breastmilk, per this healthcare provider from Nakuru: *“The pregnant women will first be worried about the potential side effects from the vaccine, and the lactating mothers—almost all of them will ask will it affect the child, will it affect breastfeeding. Some will say that when you take a certain vaccine that there is that reduction of breast milk. So I think they would ask those questions about this new vaccine.”* (Naivasha Sub-County Hospital, Nakuru).

Additional questions healthcare providers thought pregnant and lactating people would raise were related to duration of protection, dosing, and why the pregnant woman was being targeted for vaccination, as illustrated by this healthcare provider from Mombasa: *“Is it a lifelong, how long, is it lifelong, once you get it once, with subsequent pregnancies, will I still need to get the vaccine? How will I benefit from the RSV vaccine and how about my husband and the other children or the other family members, why is it only me getting the vaccine?*” (Tudor Sub-County Hospital, Mombasa). This healthcare provider from Mombasa also raised dosing: “*Is it a repeated vaccine or is it a single vaccine—what is the dosage?*” (Miritini Health Centre, Mombasa).

## Discussion

Community sensitization emerged as the most critical lesson learned from the COVID-19 vaccine rollout to inform future RSV vaccine rollout in our study. We categorized community sensitization into three domains: communication, mobilization, and education. Community sensitization included communication related to awareness of RSV and vaccines, mobilization related to building trust with communities through local trusted messengers, and education related to allaying concerns related to safety, duration of protection, and dosing.

Our study results are supported by other study findings from the region related to COVID-19 vaccine uptake among the general population [14, 16]. A barrier to COVID-19 vaccination uptake in sub-Saharan Africa was limited community sensitization when compared with other mass vaccination campaigns [17]. Given the rapid rollout of COVID-19 vaccines, community sensitization was conducted rapidly and selectively, in comparison to how community sensitization unfolds related to other vaccines [18]. In a review that examined challenges and lessons learned related to COVID-19 vaccine rollout in 22 countries in Africa, all countries recommended improved communication as a key lesson learned for future vaccine introduction [19]. Burgess et al. (2021) outlined the importance of community mobilization by supporting the development of community networks, including leveraging messengers that influence decision making, such as community and faith leaders, teachers, sports and youth clubs, and online communities and networks to build trust in communities for COVID-19 vaccine acceptance [16].

Related to pregnant and lactating women specifically, a study that examined COVID-19 vaccine acceptance among pregnant women found that communication about the disease and education related to safety concerns in Cameroon positively influenced acceptance [20]; findings were similar among a study conducted among pregnant women living in South Africa [21]. A scoping review of determinants of COVID-19 vaccine decision-making among pregnant women in sub-Saharan Africa highlighted the importance of mobilization—including engaging with community health workers, community influencers, and faith leaders to serve as channels of information and education [14]. Specific to Kenya, in a study exploring the COVID-19 vaccine decision-making process among pregnant and lactating women in Kenya, social norms related to vaccination affected the decision-making process, and authors suggested engaging with social networks and family members for targeted community mobilization [22]. Related to COVID-19 vaccine hesitancy among pregnant and lactating women in Kenya, effective messaging through education at the community level for the general population and specific to pregnant/postpartum women was identified as key to allay vaccine concerns [23]. Finally, in a study exploring COVID-19 vaccination coverage in Kenya, communication campaigns on the disease risk and importance of vaccinations were identified as successful strategies [24].

Given our study findings and from the broader literature, thinking strategically about how to plan for comprehensive community sensitization—including communication, mobilization, and education—is essential for any future vaccine. Given the sensitivity around maternal vaccines and their safety, we suggest even more extensive and concerted efforts to start sensitizing communities well before a vaccine is available. With any new vaccine, individuals will have questions, so leveraging trusted community voices is also critical. As vaccines do not save lives, but vaccination does, sensitization is crucial for future maternal vaccine acceptance.

This study has limitations. This qualitative study was not designed to be generalizable, and social desirability bias is likely. We also asked healthcare providers to discuss rollout of a vaccine that is not yet available. In addition, there are important differences between COVID-19 vaccines and the RSVpreF vaccine with respect to evaluation in randomized controlled clinical trials: COVID vaccines were developed for universal administration and testing during pregnancy was unfortunately not initiated prior to vaccine licensure and registration, whereas RSVpreF was specifically designed for administration during pregnancy and was administered to more than 3600 pregnant people prior to licensure and registration [25]. The availability of pregnancy-specific data may enhance confidence in the RSVpreF vaccine for healthcare providers, pregnant persons, and other stakeholders. Despite these limitations, this study has several strengths. This is one of the first studies that have examined how lessons learned from the COVID-19 vaccine rollout for pregnant and lactating women can inform the rollout of a future maternal RSV vaccine. By interviewing healthcare providers, we were able to obtain the perspective of the group that pregnant and lactating women go to first for questions about their health care, including vaccination. We were also able to identify key questions that people would have about the maternal RSV vaccine, including why only pregnant women would receive it, as well as duration of protection and dosing. Healthcare providers identified community sensitization, including communication, mobilization, and education, as the most important lessons to inform future maternal vaccine rollout.

## Conclusions

New maternal vaccines, including an RSV maternal vaccine, are on the horizon. Healthcare providers in Kenya used their experiences of rolling out the COVID-19 vaccine to pregnant and lactating women to provide recommendations for future maternal vaccine rollout. The most prevalent lessons identified were related to community sensitization, including communication, mobilization, and education. Future studies should explore the different aspects of community sensitization that are most likely to be successful in future vaccine acceptance, through additional qualitative studies with a variety of stakeholders, including healthcare providers, but also beneficiaries and their families. These lessons related to community sensitization should be used to prepare communities for new maternal vaccines to maximize vaccine acceptance.

## Data Availability

The datasets analyzed during the current study are available from the corresponding author on reasonable request.
